# *Gasterophilus* (Diptera, Gasterophilidae) infestation of equids in the Kalamaili Nature Reserve, China

**DOI:** 10.1051/parasite/2016036

**Published:** 2016-09-05

**Authors:** Heqing Huang, Boru Zhang, Hongjun Chu, Dong Zhang, Kai Li

**Affiliations:** 1 Key Laboratory of Non-Invasive Research Technology for Endangered Species, College of Nature Conservation, Beijing Forestry University Beijing 100083 PR China; 2 Wildlife Conservation Office of Altay Prefecture Altay 836599 Xinjiang PR China

**Keywords:** *Gasterophilus*, Equids, Przewalski’s horses, Epidemiology, Differential analysis

## Abstract

We investigated infections with *Gasterophilus* spp. in three equids within the Kalamaili Nature Reserve (northern China). We conducted necropsies on 6 Przewalski’s horses (*Equus ferus przewalskii*) and 6 Mongolian wild asses (*Equus hemionus*) and administered ivermectin to 10 overwintering domestic horses to expel parasites during winter periods. All 22 equids studied (100%) were infested with *Gasterophilus* spp. and a total of 17,225 larvae were collected. These included six species: *G. haemorrhoidalis*, *G. inermis*, *G. intestinalis*, *G. nasalis*, *G. nigricornis*, and *G. pecorum*. The mean intensity of *Gasterophilus* spp. larvae was 1904 in Przewalski’s horses, 780 in Mongolian wild asses, and 113 in domestic horses. *Gasterophilu*s *pecorum* was the most abundant species in all three equids. Przewalski’s horses, a reintroduced species, had a significantly higher intensity of *Gasterophilus* spp. than the Mongolian wild ass, indicating greater susceptibility to parasites in its ancestral home.

## Abstract (fr)

Nous avons étudié les infections de *Gasterophilus* spp. chez trois équidés dans la réserve naturelle de Kalamaili (Chine du Nord). Nous avons effectué des autopsies sur 6 chevaux de Przewalski (*Equus ferus przewalskii*) et 6 ânes sauvages de Mongolie (*Equus hemionus*) et administré de l’ivermectine à 10 chevaux domestiques hivernants pour expulser leurs parasites pendant les périodes d’hiver. Tous les 22 équidés étudiés (100 %) étaient infestés par *Gasterophilus* spp. et un total de 17,225 larves ont été recueillies. Elles comprenaient six espèces : *G. haemorrhoidalis*, *G. inermis*, *G. intestinalis*, *G. nasalis*, *G. nigricornis* et *G. pecorum*. L’intensité moyenne des larves de *Gasterophilus* spp. était 1904 chez les chevaux de Przewalski, 780 chez les ânes sauvages de Mongolie et 113 chez les chevaux domestiques. *Gasterophilus pecorum* était l’espèce la plus abondante chez les trois équidés. Les chevaux de Przewalski, une espèce réintroduite, avait une intensité significativement plus élevée de *Gasterophilus* spp. que les ânes sauvages de Mongolie, ce qui indique une plus grande sensibilité aux parasites dans leur aire ancestrale.

## Introduction

The Kalamaili Nature Reserve (KNR) (latitude: 44°36′–46°00′ N, longitude: 88°30′–90°03′ E, altitude: 600–1464 m) is located in the desert steppe of Xinjiang, China. Nomadic Kazakh populations in KNR traditionally migrate 200 km northward to summer pastures every spring and return in autumn [20]. Three equid species live in the KNR: the Przewalski’s horse, the Mongolian wild ass, and the overwintering domestic horse [3].

More than 150 species of internal parasites infect horses [5]. *Gasterophilus* spp*.* are obligate parasites that infest the gastrointestinal tracts of equids, affecting the horses’ health by absorbing nutrients and secreting toxins [15, 19]. They may cause host death when the infestation is severe [4]. *Gasterophilus* spp*.* consists of nine species distributed worldwide [21]. In China, six of them are present, namely *G. haemorrhoidalis*, *G. inermis*, *G. intestinalis*, *G. nasalis*, *G. nigricornis*, and *G. pecorum* [6, 18]. All of them have been reported in wild populations of Przewalski’s horses in KNR, China [9].

The present study was carried out on Przewalski’s horses and Mongolian wild asses that died accidentally and with preserved corpses and feces of domestic horses following antiparasitic treatment during the winter. The aim of the study was to investigate the epidemiological features of *Gasterophilus* spp*.* in the three equid species.

## Materials and methods

### Study area

**Figure 1.**
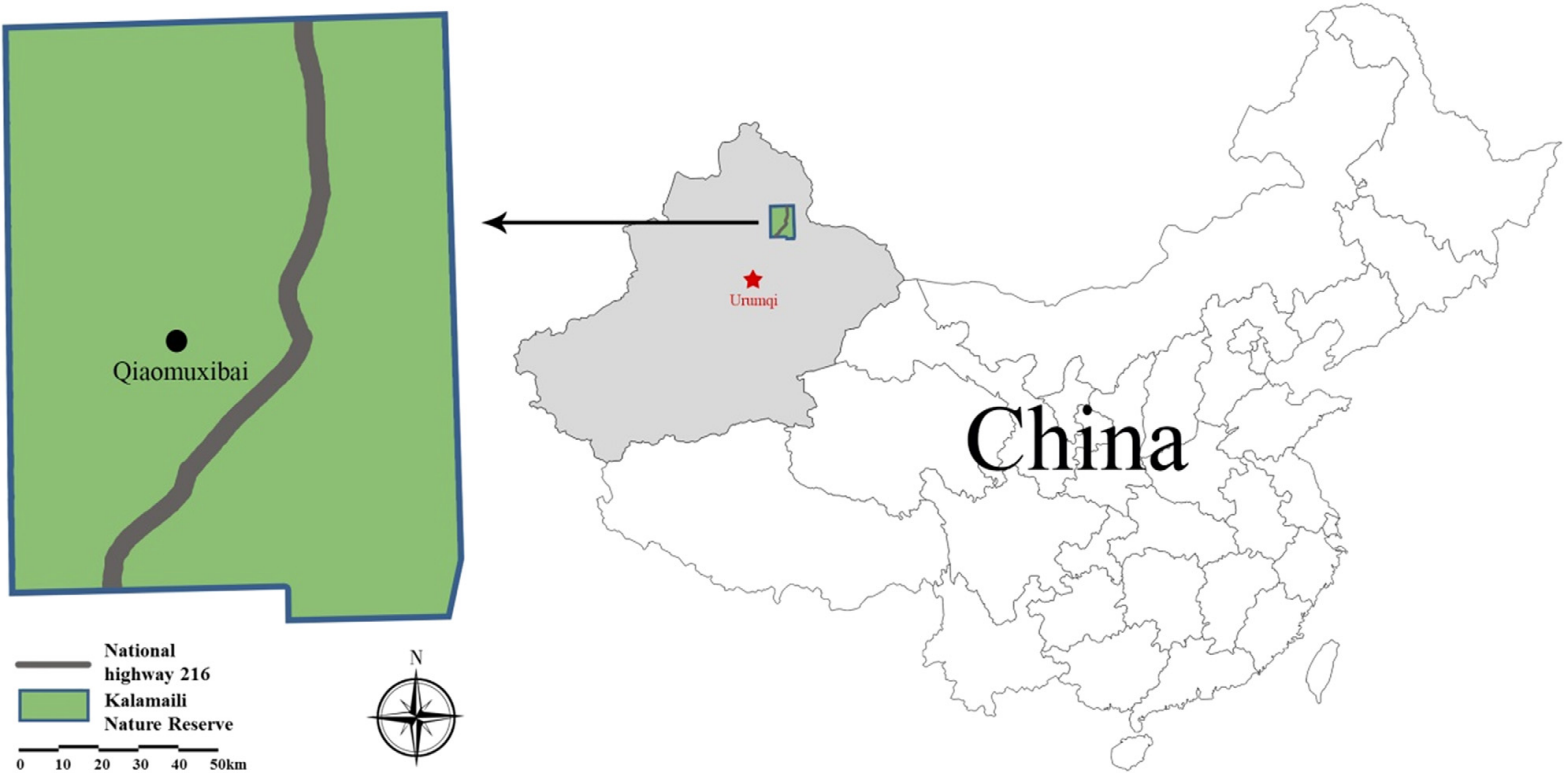
Location of the Kalamaili Nature Reserve, China.

The KNR is located in the southeast corner of the northeast Junggar Basin, Xinjiang (Fig. 1). It is dry and cold in winter and hot during the summer. Mean annual precipitation is 159 mm, and mean annual evaporation 2090 mm, which is characteristic of a typical temperate continental arid climate [2].

### Larvae collection

Six Przewalski’s horses and six Mongolian wild asses that died accidentally during the winter from 2010 to 2015 were preserved and necropsied. All *Gasterophilus* spp. larvae were collected from the digestive tracts.

A total number of 10 domestic horses (with no repeat samples) were randomly selected and treated once with ivermectin at the conventional dose of 0.2 mg/kg orally during the winter from 2013 to 2015. Following ivermectin treatment, we collected *Gasterophilus* spp. larvae that were eliminated in all feces of each horse three times a day until there were no larvae for three consecutive days. The domestic horses had received no other anti-*Gasterophilus* drug treatment prior to the survey.

Larvae were stored in ethanol (100%), washed with phosphate-buffered saline (PBS) buffer or saline solution (0.9% NaCl), counted, and identified using the morphological keys in Zumpt [21].

### Statistical analysis

The infestation prevalence, intensity, and abundance intensity were estimated according to Margolis and Schad [11].

Differences among the mean intensities of *Gasterophilus* spp. in the three equids were tested by the Kruskal-Wallis test. The statistical analysis and graphics were performed using SPSS version 20.0. A significant difference was assumed when *p* ≤ 0.05.

## Results

### Prevalence of *Gasterophilus* spp. larvae

*Gasterophilus* spp. larvae were found within all 22 individuals of all three equid species. These included six species: *G. haemorrhoidalis*, *G. inermis*, *G. intestinalis*, *G. nasalis*, *G. nigricornis*, and *G. pecorum.*

*Gasterophilus pecorum* was the most common species (100%) in the Przewalski’s horse and the Mongolian wild ass. *G. pecorum*, *G. nasalis*, and *G. nigricornis* were found in every domestic horse (Table 1).

**Table 1.** Prevalence of *Gasterophilus* spp. larvae in three equids in Kalamaili Nature Reserve, China.

*Gasterophilus* spp.	Equidae		
	*Equus ferus przewalskii*	*Equus hemionus*	*Equus caballus*
	Positive number (%)	Positive number (%)	Positive number (%)
*G. pecorum*	6 (100)	6 (100)	10 (100)
*G. nasalis*	3 (50)	4 (66.67)	10 (100)
*G. nigricornis*	2 (33.33)	4 (66.67)	10 (100)
*G. intestinalis*	2 (33.33)	1 (16.67)	7 (70)
*G. haemorrhoidalis*	3 (50)	6 (100)	7 (70)
*G. inermis*	0 (0)	1 (16.67)	3 (30)
Total	6	6	10

### Intensity infestation by *Gasterophilus* spp. larvae

A total of 17,225 larvae were collected from the three equid species. There were significant differences in the intensities of *Gasterophilus* spp. (*p* = 0.000), *G. haemorrhoidalis* (*p* = 0.047), *G. nasalis* (*p* = 0.017), and *G. pecorum* (*p* = 0.000) in the three equids. The Przewalski’s horse had the highest mean intensity of *Gasterophilus* spp. (1904) followed by the Mongolian wild ass (780) and the domestic horse (113) (*p* = 0.000) (Table 2).

**Table 2.** Intensity of *Gasterophilus* spp. larvae in three equids in Kalamaili Nature Reserve, China.

*Gasterophilus* spp.	Equidae		
	*Equus ferus przewalskii*	*Equus hemionus*	*Equus caballus*
	No.	No.	No.
*G. pecorum*	11,252	4493	621
*G. nasalis*	91	16	304
*G. nigricornis*	45	107	126
*G. intestinalis*	6	1	38
*G. haemorrhoidalis*	28	58	30
*G. inermis*	0	2	7
Total	11,422	4677	1126
Mean ± SD	1904 ± 536	780 ± 248	113 ± 54

### Abundance intensity of *Gasterophilus* spp. larvae

*G. pecorum* was the most abundant species of *Gasterophilus* spp. in the three equids. The abundance intensity of *G. pecorum* was high, especially in the Przewalski’s horse (1875.33) and the Mongolian wild ass (749.33). In the Mongolian wild ass, other species were *G. nigricornis* (17.83), *G. haemorrhoidalis* (9.67), *G. nasalis* (2.67), *G. inermis* (0.33), and *G. intestinalis* (0.17). In the domestic horse, the abundance intensity of *G. nasalis* (30.40) was higher than in the other two equids (Table 3).

**Table 3.** Abundance intensity of *Gasterophilus* spp. larvae in three equids in Kalamaili Nature Reserve, China.

*Gasterophilus* spp.	Equidae		
	*Equus ferus przewalskii*	*Equus hemionus*	*Equus caballus*
	No.	No.	No.
*G. pecorum*	1875.33	749.33	62.10
*G. nasalis*	15.17	2.67	30.40
*G. nigricornis*	7.50	17.83	12.60
*G. intestinalis*	1.00	0.17	3.80
*G. haemorrhoidalis*	4.67	9.67	3.00
*G. inermis*	0.00	0.33	0.70

## Discussion

All three equids were infected with *Gasterophilus* spp. larvae. This may reflect a wide distribution of *Gasterophilus* spp. in KNR. The high prevalence of *Gasterophilus* spp. larvae (100%) in the three equids is comparable to that reported for horses in Kazakhstan (100%) [8]. In contrast, this prevalence is much higher than reported in Sanliurfa, Turkey (9.82%) [7].

The mean intensities of *Gasterophilus* spp. larvae in Przewalski’s horses (1904) and Mongolian wild asses (780) were higher than some reports from donkeys and horses in Asia [7, 12]. The lack of anti-parasitic treatment in wild equids in KRN may be the main reason for this situation, but it may also be due to the different climate and environment. However, the domestic horse had the lowest intensity of *Gasterophilus* spp. compared to the other two equids. This difference was partly attributed to the difference of habitats between the domestic horse and the other two equids. The summer pastures were mountain meadow with a lower temperature. The vegetation and water resources were more abundant than in KRN. These different environmental conditions may affect the activity of *Gasterophilus* spp. On the whole, our results indicate that the equids in KNR are severely affected by *Gasterophilus* spp. and the winter is not the main infection period for *Gasterophilus* spp. here.

*G. pecorum* was the most abundant species of *Gasterophilus* spp. in the three equids, which differs from studies in other regions of the world where it was reported that *G. nasalis* and *G. intestinal*is are the abundant species [8, 13, 14, 16]. Our results suggest that *G. pecorum* is more adaptable to the local environment in KNR. *G. pecorum* was the only *Gasterophilus* spp. species that oviposits on grass [21]. The association with this unique behavior and the desert steppe ecosystem may help explain the situation.

Water availability restricts the activity area of wild animals in a territory such as KNR which has low precipitation, high evaporation, and limited surface runoff. A previous study showed that the oviposition sites of *G. pecorum* are often near a water source [10]. This suggests that the water locations may be the important “epidemic” areas of *G. pecorum*. Przewalski’s horses seem to drink daily [17]. For wild asses, it is often assumed that they can “regularly do without water” [1]. Frequent drinking at water sources may increase the risk of *G. pecorum* infection. Thus, the equids in arid desert grasslands have a higher intensity of *Gasterophilus* spp., and the intensity in Przewalski’s horses is higher than in Mongolian wild asses.

## References

[1] Bahloul K, Pereladova OB, Soldatova N, Fisenko G, Sidorenko E, Sempere AJ. 2001 Social organisation and dispersion of introduced kulans (*Equus hemionus kulan*) and Przewalski horses (*Equus przewalskii*) in the Bukhara Reserve, Uzbekistan. Journal of Arid Environments, 47, 309–323.

[2] Chu HJ, Jiang ZG, Lan WX, Wang C, Tao YS, Jiang F. 2008 Dietary overlap among kulan *Equus hemionus*, goitered gazelle *Gazella subgutturosa* and livestock. Acta Zoologica Sinica, 54(6), 941–954 (in Chinese).

[3] Chu HJ, Jiang ZG, Ge Y, Jiang F, Tao YS, Wang C. 2009 Population densities and number of khulan and goitred gazelle in Mt. Kalamaili Ungulate Nature Reserve. Biodiversity Science, 17(4), 414–422 (in Chinese).

[4] Czosnek T. 1988 *Gasterophilus* infestation, the cause of death in a mare. Medycyna Weterynaryjna, 44, 346.

[5] Doyle GM, John EH, Craig RR. 2003 Control of internal parasites of the horse. University of Tennessee, Institute of Agriculture, Website: http://trace.tennessee.edu/utk_agexani/34/. Accessed 19 December 2015.

[6] Fan ZD. 1992 Index of common flies in China. Science Press: Beijing p. 890–895 (in Chinese).

[7] Gökcen A, Sevgili M, Altas MG, Camkerten I. 2008 Presence of *Gasterophilus* species in Arabian horses in Sanliurfa region. Türkiye Parazitoloji Dergisi, 32, 337–339. 19156607

[8] Ibrayev B, Lider L, Bauer C. 2015 Gasterophilus spp. infections in horses from northern and central Kazakhstan. Veterinary Parasitology, 207(s1–2), 94–98. 25522954 10.1016/j.vetpar.2014.11.015

[9] Li K, Wu Z, Hu DF, Cao J, Wang C. 2007 A report on new causative agent (*Gasterophilus* spp.) of the myiasis of Przewalski’s horse occurred in China. Acta Veterinaria et Zootechnica Sinica, 38(8), 837–840 (in Chinese).

[10] Liu SH, Hu DF, Li K. 2014 Oviposition site selection by *Gasterophilus pecorum* (Diptera: Gasterophilidae) in its habitat in Kalamaili Nature Reserve, Xinjiang, China. Parasite, 22, 34. 26621549 10.1051/parasite/2015034 PMC4664853

[11] Margolis L, Schad GA. 1982 The use of ecological terms in parasitology (report of an ad hoc committee of the American Society of Parasitologists). Journal of Parasitology, 68(1), 131–133.

[12] Mukbel R, Torgerson PR, Abo-Shehada M. 2001 Seasonal variations in the abundance of *Gasterophilus* spp. larvae in donkeys in northern Jordan. Tropical Animal Health and Production, 33(6), 501–509. 11770204 10.1023/a:1012732613902

[13] Otranto D, Milillo P, Capelli G, Colwell DD. 2005 Species composition of Gasterophilus spp. (Diptera, Oestridae) causing equine gastric myiasis in southern Italy: parasite biodiversity and risks for extinction. Veterinary Parasitology, 133(1), 111–118. 15978726 10.1016/j.vetpar.2005.05.015

[14] Pandey VS, Ouhelli H, Verhulst A. 1992 Epidemiological observations on *Gasterophilus intestinalis* and *Gasterophilus nasalis* in donkeys from Morocco. Veterinary Parasitology, 41(3–4), 285–292. 1502790 10.1016/0304-4017(92)90087-p

[15] Principato M. 1988 Classification of the main macroscopic lesions produced by larvae of *Gasterophilus* spp. (Diptera:Gasterophilidae) in free-ranging horses in Umbria. Cornell Veterinarian, 78(1), 43–52. 3335129

[16] Principato M. 1989 Observation on the occurrence of five species of *Gasterophilus intestinalis* larvae in free-ranging horses in Umbria, Central Italy. Veterinary Parasitology, 31(2), 173–177. 2741303 10.1016/0304-4017(89)90032-0

[17] Scheibe KM, Eichhorn K, Kalz B, Streich WJ, Scheibe A. 1998 Water consumption and watering behavior of przewalski horses (*Equus ferus przewalskii*) in a semireserve. Zoo Biology, 17, 181–192.

[18] Wang MF 1998 *Gasterophilus* spp., in Flies in China (volume II), Science and Technology Press: Shenyang p. 2207–2215 (in Chinese).

[19] Yang JY, Zhang D, Hu DF, Chu HJ, Tao YS, Fan XZ, Li K. 2013 The injury caused by myiasis of *Gasterophilus* in horse. China Animal Husbandry and Veterinary Medicine, 40(5), 177–180 (in Chinese).

[20] Yi Y, Song DR. 2008 Transitions: the prairie of Kazak herders and the stipulations. China Mapping, 1, 58–65 (in Chinese).

[21] Zumpt F. 1965 Myiasis in man and animals in the old world: a textbook for physicians. Veterinarians and Zoologists: Butterworth, London.

